# Advancing Glass Engineering: Harnessing Focused Electron Beams for Direct Microstructuring

**DOI:** 10.1002/smtd.202401671

**Published:** 2025-03-09

**Authors:** Mathias Holz, Martin Hofmann, Christoph Weigel, Steffen Strehle

**Affiliations:** ^1^ Technische Universität Ilmenau Institute of Micro‐ and Nanotechnologies Microsystems Technology Group Max‐Planck‐Ring 12 98693 Ilmenau Germany

**Keywords:** direct structuring, electron beam, glass, microstructuring

## Abstract

A technological approach for direct glass structuring is presented by exploiting electron‐beam‐induced defect generation utilizing a conventional scanning electron microscope (SEM). The structuring process is assumed to be linked to electron‐beam‐induced ion migration and allows to create structures of several hundred nanometers in depth. It is demonstrated that the structuring can be realized in literally any SEM, which thus enables a comparatively simple implementation in support of a broad field of applications. The experiments are realized using electron energies of 5 to 15 keV in combination with different kinds of glasses, such as fused silica and ultra‐low expansion glass, that are equipped with a charge dissipation top‐layer. By controlling the beam trajectory at the surface and the electron beam parameters, freeform structuring, structure arrays, direct embedding of metal structures into the glass surface, and beam‐defined three‐level patterning are realized. The shown electron beam‐based glass structuring extends therefore the current possibilities in a complementary manner, enabling further fabrication strategies and direct structuring even of fragile, 3D‐structured surfaces.

## Graphical abstract

An approach for the direct patterning of glasses is presented, using electron‐beam‐induced defect generation in a conventional scanning electron microscope. This method enables the direct microstructuring of glasses with charge‐dissipation top‐layers, such as fused silica, using electron energies of 5–15 keV. Applications include freeform patterning, metal embedding, and multi‐level patterning, offering new fabrication possibilities even for 3D‐structured glass surfaces.

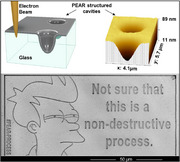

## 1 Introduction

Since its introduction, SEM has been recognized as an extremely valuable and, above all, mainly non‐destructive technique for surface analysis of inorganic materials. In addition to microscopy, focused electron beams are also used in electron beam lithography to create in combination with subsequent post‐treatments, for example, nanostructures in electron‐sensitive resists.^[^ ^1^ ^]^ For direct nanofabrication of micro‐ and nanoscale structures, special gaseous precursors can be decomposed locally on a substrate surface in interaction with a focused electron beam.^[^ ^2^, ^3^ ^]^ In addition to these established techniques, we demonstrate in this paper that focused electron beams, as generated in a conventional scanning electron microscope, are sufficiently destructive in nature in relation to different types of glasses. This must be considered during electron microscopic sample inspections, but we also show here that this otherwise destructive effect can be used directly for the surface structuring of glass surfaces without a requirement of polymeric resists, precursor gases, or chemical post‐treatments. Inspired by the generally assumed pear‐shaped interaction volume between a focused electron beam and the material surface, the process is denoted in the following as PEAR (patterning by electron‐beam activated relocation).

**Figure 1:**
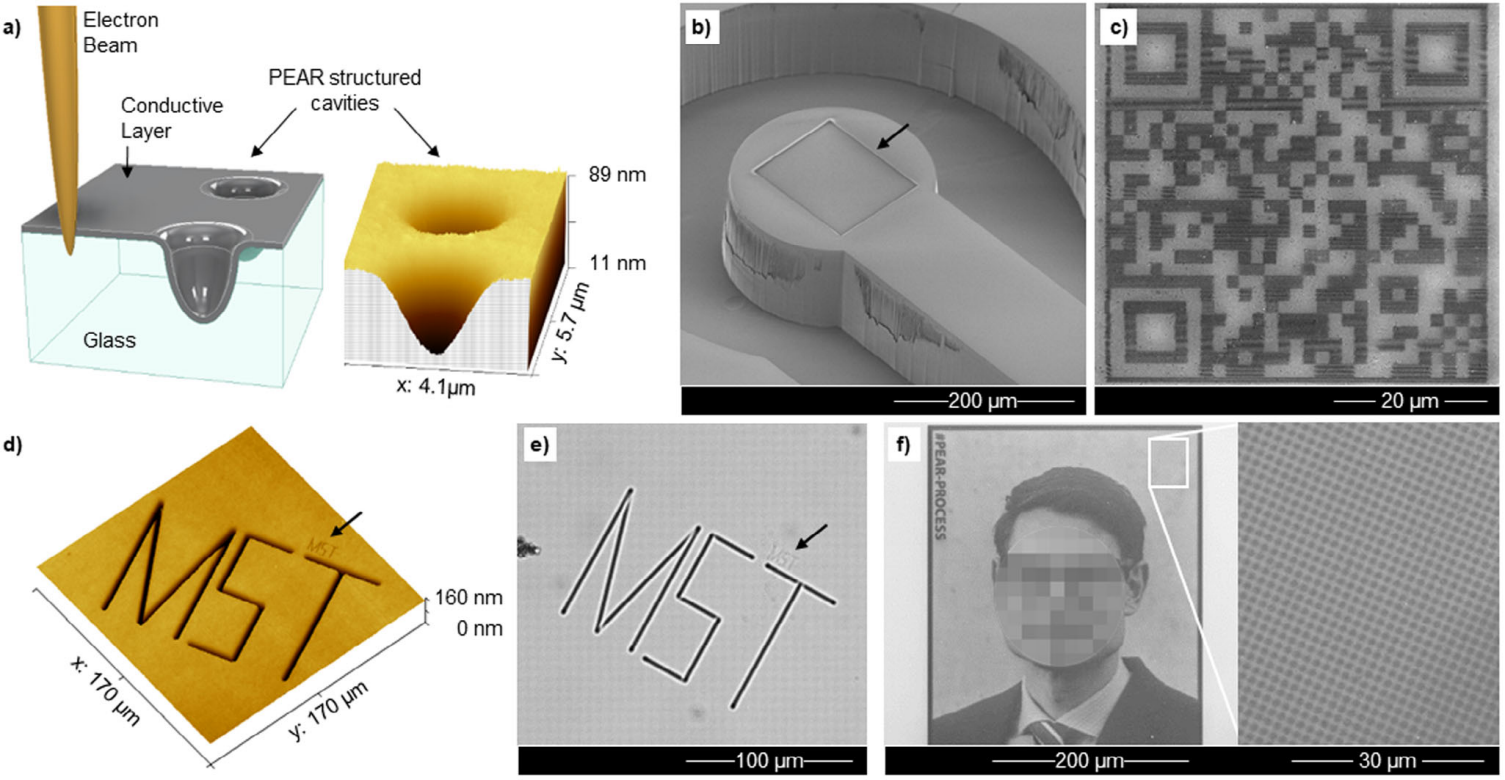
Examples for structuring using the PEAR process: a) (left) schematics of the PEAR structuring (not to scale) and (right) atomic force microscopy surface reconstruction of a PEAR cavity created in fused silica; b) PEAR structuring on a 3D‐structured micromechanical element (duration ≈1 h); c) SEM image of a QR code created by a PEAR process in ≈3 h that can be used as a security feature, for example; d) atomic force microscopy image and e) optical microscopy image of the letters “MST” (MST: Microsystems Technology) structured in two sizes (smaller one is marked with an indicator arrow) into fused silica in ≈20 min; f) SEM image of an example of a photographic halftone image produced with PEAR structuring in fused silica glass (Spectrosil2000) surface. The SEM image has been partially pixelated to protect personal rights. A 15 nm thick aluminum top layer was used (original photography: S. Günther‐Müller/TU Ilmenau).

To provide a first glimpse of PEAR and the broad range of structuring capabilities, some examples are provided in **Figure** **1**. The specific mechanisms underlying PEAR need to be still further elucidated in the future. Nevertheless, based on current knowledge of electron beam‐induced defect generation in insulating materials like glass, the following mechanisms can be discussed. First, electron‐induced surface charging and material desorption can change the local composition and cause atom and ion diffusion. Second, electron beam‐induced heating at the electron beam impact zone can also support ion migration, which is, based on literature, especially true for mobile ions that are even affected at greater depths (between 0.5–2 µm).^[^ ^4^ ^]^ Third, electron‐induced breakage of chemical bonds and associated chemical and morphological altering can occur if the bonds are not re‐established within a certain time, which should be relatively long and expected in the microsecond range for non‐conductive materials.^[^ ^5^ ^]^

Although our findings, at least partially, challenge the often‐assumed non‐destructive nature of scanning electron microscopy, they provide simultaneously complementary possibilities for microfabrication that add strategies for various application scenarios in the field of glass microstructuring (cf. Figure 1). Glasses are present as emerging high‐performance materials in various fields with direct ties to modern micro‐, nano‐, and biotechnology, with numerous applications directly benefiting from functional micro‐ and nanostructuring. Such applications were, for instance, demonstrated for optics,^[^ ^6^, ^7^ ^]^ medical technology,^[^ ^7^, ^8^, ^9^ ^]^ nano‐ and micromechanical systems,^[^ ^10^, ^11^, ^12^ ^]^ chip packaging,^[^ ^13^ ^]^ precision metrology,^[^ ^14^ ^]^ construction,^[^ ^15^ ^]^ and even arts and crafts.^[^ ^16^ ^]^

Glass pattering itself is therefore well known. Established wet chemical etching of glasses, for instance, with hydrofluoric acid, requires adequate surface masking and yields a distinct isotropic etch profile.^[^ ^17^ ^]^ The need for appropriate surface masking also applies to reactive ion etching, which however, can be used to produce optical‐quality micro‐ and nanostructures even with near‐vertical sidewalls, depending on the plasma chemistry used and the type of glass.^[^ ^18^, ^19^ ^]^ To avoid elaborate surface masking, which is associated with the risk of surface contamination and mechanical stress, direct structuring processes are intriguing. Laser ablation, as one prominent example of a direct glass structuring process, is limited in its patterning resolution typically in the micrometer range, and can in addition also impair unstructured regions.^[^ ^20^, ^21^ ^]^ Further methods like reactive atom plasma etching,^[^ ^22^, ^23^ ^]^ femtosecond laser‐induced structuring^[^ ^24^ ^]^ or ion beam machining^[^ ^25^, ^26^ ^]^ represent attractive alternatives particularly for micro‐ and nanoscale applications. They can also induce unwanted structural changes within the material, if the energy impact is too high^[^ ^24^, ^27^ ^]^ or necessitate certain post treatments.^[^ ^28^ ^]^ Without seeking to replace any existing technology, we show that PEAR structuring adds possibilities to directly structure glass using focused electron beams that can be generated already in a conventional SEM.

The structures produced by PEAR are optically and mechanically detectable (cf. Figure 1). To rationally control PEAR structuring already at this early stage, we explored the influence of the electron beam current, the electron acceleration voltage (5 to 15 keV), and the kind and thickness of the utilized conductive charge dissipation top layer, as discussed in detail in the following. In brief, based on our findings, we were able to determine a parameter set for patterning and one for SEM imaging with the least surface degradation. Direct PEAR structuring was studied in several SEMs and realized so far for eight types of glass substrates including fused silica, ultra‐low expansion glass, as well as boro‐, alumo‐ and titanium‐silicate glass. The PEAR structuring as well as required SEM inspections were experimentally carried out mainly in a Helios 600i from FEI but were also successfully verified in other devices including a Zeiss Auriga 60 (Figure S17, Supporting Information) and a Hitachi 4800, thus demonstrating its broader applicability. The experiments highlight the application potential of PEAR structuring for glass and glass‐ceramics as well as the associated drawbacks for standard surface inspection.

## 2 Results and Discussion

**Figure 2:**
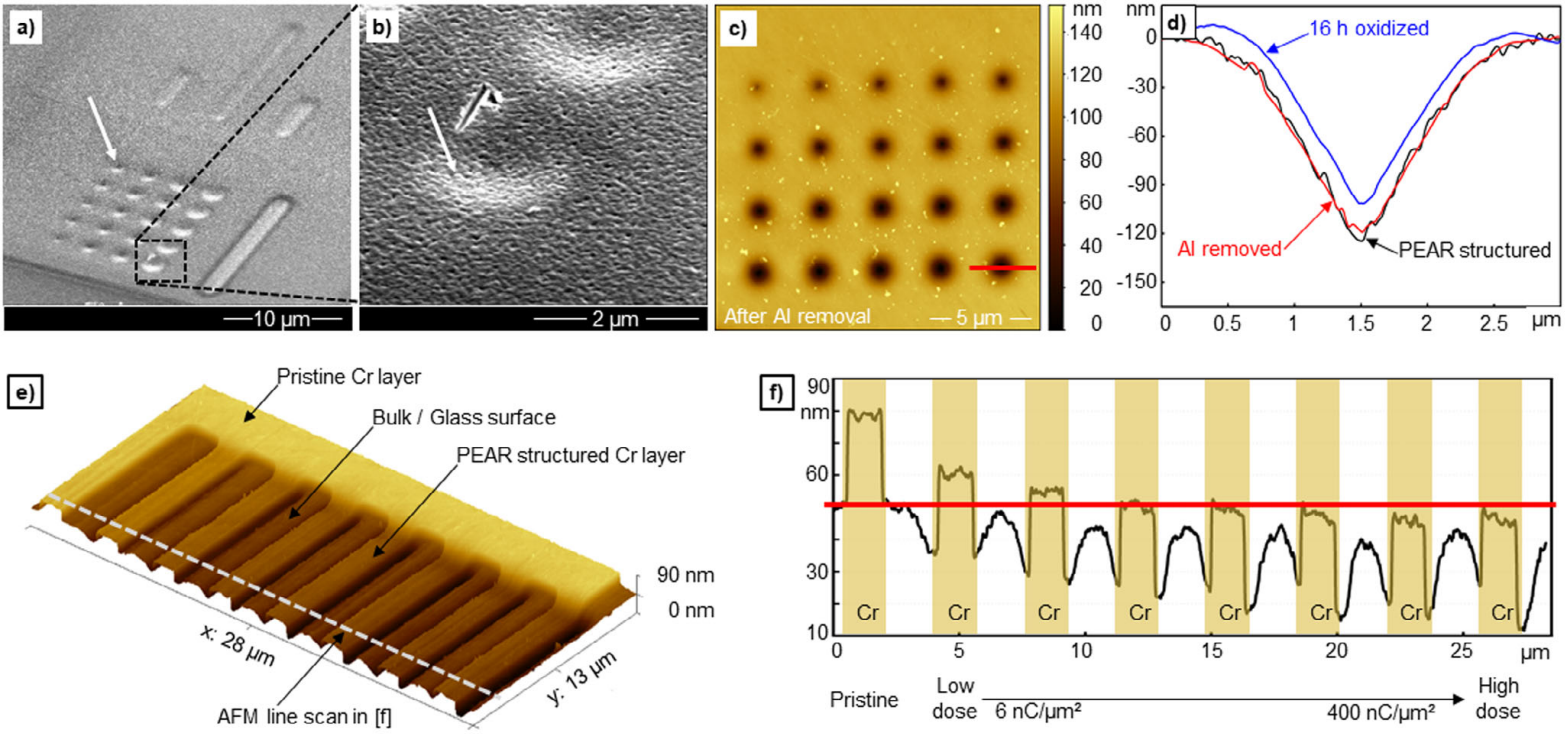
a) SEM image of PEAR structured cavity array in Spectrosil2000 with 60 nm Al conductive top‐coating (PEAR: 10 kV, 5.5 nA varied dose 15 to 930 µC µm^−^^2^); b) SEM image of a single exemplary cavity (620 µC µm^−^^2^) as marked in (a) indicating the collapse of the metal layer into the underlying PEAR structured cavity. The metal layer is deformed, but otherwise intact (see indicator arrow). The AFM induced scratch next to structure shows actual metal removal; c) AFM surface scan of the circular cavity pattern from (a) after removal of the 60 nm Al conductive coating; d) Line profiles (930 µC µm^−^^2^) as marked in (c) measured with AFM prior and subsequent to Al stripping and sample oxidation; e) Demonstration of direct metal embedding into the glass surface. An electrode comb structure (30 nm thick chromium) on Spectrosil2000 was used. The metal lines were exposed from 6 to 397 nC µm^−^^2^ to create varying embedding depths during PEAR structuring. f) AFM line scan as indicated in (e) illustrating the metal line embedding. Coplanar embedding is approximately reached at the fifth line, which corresponds to a dose of 50 nC µm^−^^2^.

For PEAR structuring, the glass substrate surfaces were equipped with an ≈5 to 100 nm thin, electrically conductive top layer consisting of carbon, aluminum, chromium, tungsten, and gold. Such conductive top layers are commonly deposited onto insulating materials for SEM inspection to minimize surface charging effects that would otherwise impair the electron beam focus and thus the imaging quality. However, since the penetration depth of the electron beam is typically much greater than the thickness of the conductive surface layer, electrons are also injected into the material underneath this layer.^[^ ^29^, ^30^ ^]^ This conductive surface layer also hinders material from leaving the glass surface, which means that PEAR structuring should be hardly based on the generation of volatile reaction products like oxygen. However, it must first be ensured that the conductive surface layer remains intact and that PEAR actually takes place in the glass and not in the upper metal layer itself. Komissarenko et al.,^[^ ^31^ ^]^ for instance, reported the formation of microstructures within gold and aluminum films on glass due to electron beam irradiation. In contrast, we were able to observe a different effect. As exemplarily shown in **Figure** **2**a,b for synthetic fused silica glass (Spectrosil2000) covered by a chromium/aluminum film stack (nominal thickness: 10/50 nm), the metal top layer appears intact. Visual inspection by means of SEM could not reveal any cracking, swelling, or ablation, which was also true for all inspected samples. The top layer collapses into a cavity that was created underneath the glass surface. Furthermore, atomic force microscopy (AFM) surface inspections of the same structures recorded after PEAR structuring before and after wet‐chemical removal of the conductive metal top layer confirmed within the range of errors that the cavities are indeed created in the glass (Figure 2c). Lineweaver^[^ ^32^ ^]^ reported an outgassing of oxygen from glass samples when exposed to an electron beam of 150 µA at 20 kV for several h. Although especially a 60 nm thick chromium/aluminum top layer should effectively prevent an outgassing of oxygen from the glass by mechanical and chemical blocking, the effect must be still considered in principle as a reason for the cavity formation in the glass surface. O'Keeffe and Handy^[^ ^33^ ^]^ reported in this regard that the glass degradation caused by electron bombardment can be completely reversed by annealing samples in an oxygen‐rich atmosphere (e.g., 800 °C in the air for 30 min). Nevertheless, annealing in an oxygen‐rich atmosphere and temperatures of up to 1050 °C for 16 h resulted in our samples only a minute surface change, but hardly in a restoration of the cavities created (Figure 2d). Areas that underwent PEAR structuring did also not differ significantly in chemical composition, e.g. lower oxygen concentration, which was explored by X‐ray photoelectron spectroscopy on a PEAR structured 1 × 1 mm^2^ field. These observations currently rule out outgassing as a significant contribution to cavity formation in favor of internal material migration and displacement effects. Furthermore, Raman spectroscopy measurements were done on pristine and PEAR structured surfaces of the fused silica glass (Spectrosil2000) after removal of the conductive top layer (Figure S16, Supporting Information). Here it becomes evident that PEAR structured areas show a distinct characteristic peak (D2) increase at 605 1/cm that is frequently associated with changes in the glass network, such as 3‐member silicon‐oxide ring formation.^[^ ^24^, ^34^, ^35^, ^36^, ^37^ ^]^

The fact that the metal top layer stays intact during PEAR structuring but sinks into the glass surface can be exploited as a possibility to directly embed metal structures in a glass surface. For demonstration purposes, we created an arrangement of chromium metal lines (thickness 30 nm) rather than a closed film and exposed the lines differently with 6 to 397 nC µm^−^^2^ of electron bombardment, which is expected to yield different embedding depths. As clearly shown in Figure 2e,f, the embedding depth can be indeed controlled. While for a dose of 12 nC µm^−^^2^ the metal surface still stands out by ≈5 nm, planarity between glass and metal surface is approximately reached at a dose of ≈50 nC µm^−^^2^. At larger doses, the metal surface is below the surrounding glass surface. These experiments demonstrate again that the glass surface rather than the metal is patterned and furthermore, that the patterning depth for PEAR structuring can be controlled.

For the parametric evaluation of PEAR structuring, a standard test pattern was designed. The dose pattern consisted of a series of circular electron beam exposure regions with a diameter of 20 nm (cf. Figure 2a indicator arrow), which were exposed with 15 to 930 µC µm^−^^2^ (cf. Figure 5a). For a given beam current, the dose is defined by the exposure time as shown in the Methods section, Figure 5. For example, if the beam current is set to 5.5 nA, a dose of 15 µC µm^−^^2^ will give 1 s and a dose of 930 µC µm^−^^2^ will result in 60 s exposure time.

**Figure 3:**
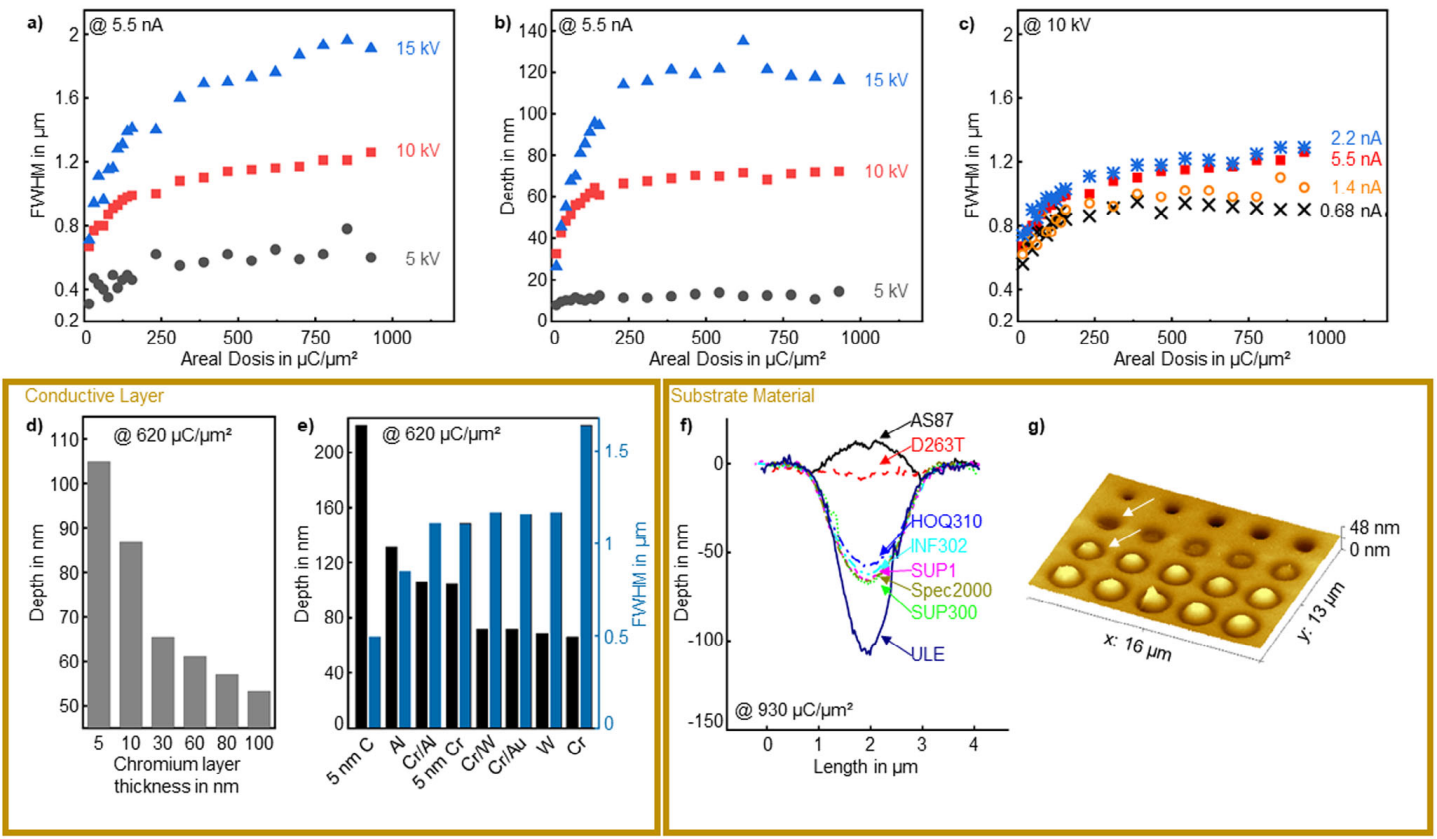
a–c) Dependence of the cavity diameter and depth (examined by AFM) on the used acceleration voltage and beam current. PEAR structuring was realized on Spectrosil2000 covered with 10/50 nm Cr/Al conductive top coating using a dose from 15 to 930 µC µm^−^^2^. For (a,b) a constant beam current of 5.5 nA and for (c) a constant acceleration voltage of 10 kV was used; d) PEAR structured cavity depth depending on a Cr top layer thickness using a constant dose of 620 µC µm^−^^2^; e) PEAR cavity depth and FWHM (620 µC µm^−^^2^) for different kinds of conductive top layer materials (60 nm total layer thickness, if Cr‐based stacks are used, Cr is 10 nm in thickness); f) AFM line profiles showing the dependence of the kind of substrate material on PEAR structuring (930 µC/µm^2^ at 10 kV and 5.5 nA, 30 nm conductive Cr top‐coating); g) AFM surface scan of the standard test pattern created by PEAR structuring in the glass AS87 illustrating hillock formation for higher doses than 232 µC µm^−^^2^ (lower indicator arrow). Lower doses, e.g. of 93 µC µm^−^^2^ (upper indicator arrow), yield however the aforementioned PEAR cavities.

A key parameter in SEM, which is assumed to be equally true for PEAR structuring based on the initial model of a charging underneath the conductive top layer, is the electron acceleration voltage that defines, for instance, the depth that the electrons can penetrate a material. To remain within the usual operating parameters of common SEMs, 5, 10, and 15 keV were selected as electron acceleration voltages for a systematic evaluation with respect to PEAR structuring. A constant beam current of 5.5 nA and again synthetic fused silica (Spectrosil2000) covered with a chromium/gold film stack (thickness: 10/50 nm) were used for the evaluation. The standard test patterns created by PEAR structuring were characterized by AFM for each set acceleration voltage in terms of the created cavity diameters, expressed as the full width at half maximum (FWHM) of the cavity cross section profile and in terms of the created central cavities depth. As shown in **Figure** **3**a,b, the PEAR structured cavities tend to widen and deepen with increased acceleration voltage regardless of the set dose. PEAR structuring realized with electrons accelerated with 15 kV and a dose of 930 µC µm^−^^2^ yield, for example, ≈115 nm deep cavities with an FWHM diameter of 1.91 µm. Although the PEAR cavity aspect ratio is still low, the structures can be already used for certain applications as discussed before. Lowering the acceleration voltage to 5 kV lowers accordingly the achieved depth to ≈15 nm and the FWHM diameter to 0.60 µm. The distinct dependence of the cavity diameter and depth on the acceleration voltage indicates that the electron‐material interaction volume that is defined by the kinetic electron energy, aside from other parameters, is a key parameter for PEAR structuring.

Based on our initial model assumptions for PEAR structuring including electron‐induced surface charging, material desorption, electron‐beam‐induced heating and electron‐induced breakage of chemical bonds, a direct correlation to the number of injected electrons into the glass and therefore, to the dose can be also assumed. The achieved cavity depth rises indeed clearly in accordance with the electron dose but only up to ≈200 µC µm^−^^2^. For higher doses, the depth and diameter of the cavities tend to saturate regardless of the selected acceleration voltage as shown in Figure 3a,b.

This saturation effect suggests a charge saturation in the material that scatters and deflects the electron beam in response. The rate of electron material interactions can be affected by changing the beam current, which was accordingly varied from 0.68 nA to 5.5 nA using a constant acceleration voltage of 10 kV. It is assumed that a higher beam current, associated with a shorter exposure time, suppresses internal material as well as charge relaxation and migration effects, which should therefore affect the material displacement rate. As expected, increasing the beam current and thus shortening the exposure time yields slightly wider structures (Figure 3c). Nevertheless, the overall dependence of the achieved cavity diameter and depth on the injected charge per area persists. Furthermore, for the used acceleration voltage and beam current parameters, no significant change of the cavity depth is observed, which appears therefore predominately linked to the acceleration voltage and thus to the electron kinetic energy. Parasitic carbon deposition, being well‐known for SEM, must also be discussed, which would result in a smaller FWHM and depth of the cavities. However, the fact that the depth is mostly independent of the beam current suggests that there is no significant contribution from localized beam‐induced carbon deposition for beam currents larger than 0.68 nA. However, further lowering the current for the same dose, presumed carbon deposition becomes indeed more and more a dominating effect. The presumed carbonaceous surface deposits can be achieved, for example, with 10 kV electron acceleration voltage and a beam current of 0.086 nA (writing time ≈1 h, dose 930 µC µm^−^^2^) as shown in the supplementary (Figures S2 and S3, Supporting Information). We can conclude from these experiments that these parameters can be used for SEM imaging with reduced acceleration voltage down to 5 kV while sufficiently suppressing PEAR structuring.

The primary electron kinetic energy respectively the yielded glass penetration depth is intrinsically also affected by the type and thickness of the conductive top layer. The conductive layer thickness should be in principle as thin as possible and ideally made from a light‐weight element with respect to a minimized impact on the primary electrons. We investigated therefore the role of the conductive layer by studying six different layer thicknesses ranging from 5 to 100 nm for a Cr thin film and additionally six different materials using a fixed total thickness for the conductive top‐coating of 60 nm as shown in Figure 3d,e, respectively. Figure 3e shows clearly the proposed relationship that the thinner the top layer thickness, the deeper the cavities obtained. While the achieved depth for a 100 nm thick Cr layer is less than 60 nm, almost 90 nm is, for instance, obtained if the Cr thickness is reduced to only 10 nm. This dependence on the conductive layer thickness could be potentially also exploited in the future as one strategy to control the 3D‐morphology and the aspect ratio of cavities within PEAR structuring. The cavity diameter is however not subject to any significant change (not shown). PEAR structuring appears, based on the experiments, to be limited to a maximum chromium layer thickness of ≈300 nm (for dose 930 µC µm^−^^2^) estimated here by plain linear extrapolation. Corresponding Monte Carlo simulations of electron trajectories, as shown in the supplementary (Figure S4, Supporting Information), confirm this and show that most of the electron energy is dissipated in the chromium top layer when the layer thickness is ≈300–320 nm. The underlying glass surface is therefore hardly modified anymore by the electron bombardment.

An influence of the type of conductive top coating was observed as well as suspected (Figure 3e). For aluminum, as the light‐weight conductive material, ≈130 nm deep cavities are obtained, whereas chromium yields the lowest depth among all studied materials of only ≈65 nm using otherwise the same PEAR parameters. The cavity diameter correlates inversely with the depth, which supports the assumption of a distinct contribution from electron scattering.

To explore and push the limits of PEAR structuring, a few nanometers (<5 nm) of carbon were deposited as the ultra‐thin lightweight conductive top layer. However, neither the exact thickness nor the allotropy of the carbon used is known but should not be of relevance for this first benchmarking. As expected, the largest cavity depth of ≈230 nm and the smallest cavity FWHM diameter of ≈565 nm is achieved (930 µC µm^−^^2^, 10 kV, 5.5 nA). In principle and as implemented later, such ultra‐thin carbon films could be also created directly in an SEM by using the aforementioned parameter set for imaging that is accompanied by parasitic carbon deposition. In summary, the experiments show that the depth can be controlled by the dose up to ≈200 µC µm^−^^2^, where saturation is reached, and by the acceleration voltage, as shown in Figure 3b, as well as by the type and thickness of the conductive top layer (Figure 3d,e). The process conformity described as the standard deviation is expected for PEAR structuring of Spectrosil2000 in the range of about ± 50 nm for the cavity width and for the corresponding depth in the range of about ± 5 nm (Figure S8, Supporting Information). This shows that the PEAR process can be used for nanostructuring despite its early technology stage and despite the fact that neither specialized tools nor optimized substrate materials exist yet.

To demonstrate the general usability of PEAR for glass patterning and to contribute to future strategies to develop specialized glass substrates for PEAR structuring, eight substrate materials offering a variety of chemical material compositions were evaluated. As evidently shown in Figure 3f, all fused quartz and fused silica glasses as well as the titanium silicate glass could be structured by PEAR but different depths and diameters of cavities were achieved despite the use of the same parameters set (see **Table** **1** for a detailed materials overview). The patterning depth of the fused quartz substrates (HOQ310 and INF302) is, for instance, slightly lower compared to the synthetic fused silica substrates (Spec2000, SUP1, and SUP300), which could be linked to minute concentrations of alkaline impurities since HOQ310 and INF302 glasses were produced from natural raw materials utilizing a melting process.

**Table 1:** Application‐specific classification and characteristics of the glasses used. Further information about the glass materials with corresponding references can be seen in (Figure S13, Supporting Information).

Glass type	Trade name	Provider	Fabrication method	Properties/Applications
Fused silica	Spectrosil 2000	Heraeus	synthetic fused silica (reference)	Deep UV applications with high transmission between 180…2000 nm (OH‐content ≤ 1300 µg g^−1^)
	Suprasil 1, 300 and 312		synthetic fused silica	Optical application in UV‐Vis range (OH‐content from 1…1300 µg g^−1^)
Fused quartz glass	HOQ 310		natural fused quartz	Cost‐effective for simple optical applications (OH‐content 30 ppm µg g^−1^)
	Infrasil 302		natural fused quartz	1D IR‐optics (OH‐content ≤ 8 µg g^−1^)
Borosilicate glass	D 263 T	Schott	melting process of natural raw materials	Imaging, sensor and semiconductor technology, RF/HF applications
Alumosilicate glass	AS 87 eco		melting process of natural raw materials	High‐strength thin glass for sensor and display protection
Titanium silicate glass	ULE 7972	Corning	Synthetic glass	Ultra‐low thermal expansion applications (e.g., geometric reference structures)

In addition, PEAR structuring can also be used to structure silicon dioxide films rather than bulk substrates, for instance, in the form of silicon dioxide top layers fabricated by thermal oxidation of silicon (Figure S11, Supporting Information) and as part of silicon‐on‐insulator substrate (Figure S12, Supporting Information). Thus, PEAR structuring is also in this regard aligned with microelectronic fabrication needs. In comparison to silica, titanium silicate ultra‐low expansion glass (ULE) showed a significant increase in the depth of the PEAR cavities, which is currently attributed to the differences in the glass network. In TiO_2_‐SiO_2_ glass compositions like ULE, titanium is tetravalently bonded and acts as a network former by replacing silicon.^[^ ^38^, ^39^ ^]^ Also for reactive ion etching, higher etch rates were found for such glass compositions (in comparison to silica), which could be attributed to a less stable glass network.^[^ ^19^ ^]^ For titanium oxide (a component of titanium silicate glasses), for example, the change from Ti^4+^ to Ti^3+^ induced by laser irradiation is discussed, which can lead to a change in coordination within the glass network or to crystalline phases at the point of irradiation.^[^ ^24^ ^]^ An influence of alkaline impurities should be excluded because this glass is produced synthetically from the gas phase.^[^ ^40^ ^]^

Besides the before shown PEAR cavity formation, the borosilicate glass D263T as well as the alumosilicate glass AS87 appear to resist PEAR cavity formation at higher doses (>232 µC µm^−^^2^). Especially for the alumosilicate glass AS87, extruded features were detected in dependence on the used dose (>232 µC µm^−^^2^, Figure 3g). These features are hereafter referred to as hillocks and appear to grow out of a shallow PEAR cavity starting at a critical dose. This leaves a characteristic annular cavity surrounding the grown hillock and indicates at least two parallel material displacement mechanisms. Gedeon et al. have found similar results for alkali‐rich glasses. A possible cause of this is the high number of dangling bonds that form Si‐O‐O‐Si units under electron radiation, which require more space and thus lead to the expansion of the material.^[^ ^41^ ^]^

Hillock formation could in principle be also associated with the well‐known and aforementioned beam‐induced carbon deposition from the SEM chamber atmosphere. However, the hillock formation phenomenon was only observed for the materials AS87 and D263T (Figure 3f,g) and only for higher doses (>232 µC µm^−^^2^), which rather indicates a material effect. Borosilicates like D263T and alumosilicates like AS87 possess a high amount of mobile alkaline ions in the glass network, which might be activated by substrate (surface) charging.^[^ ^42^ ^]^ A promoting effect through electron beam‐induced heating is conceivable.^[^ ^43^, ^44^ ^]^ The required activation energy for ion diffusion processes as well as the ionic mobilities were simulated elsewhere for alkali ions embedded in a silica glass network^[^ ^45^ ^]^ and it was found that the ionic mobility is constant with temperature and independent from the chemical composition. Therefore, charge‐dependent beam‐induced ion generation and migration are assumed here to dominate. Ion diffusion and migration from or to the surface could be therefore driving forces for PEAR structuring. Since the glass substrates have low but similar thermal conductivity and capacity, it can be assumed that a comparable local increase in surface temperature will occur (Figure S13, Supporting Information). Electric field induced ion migration in glass is known^[^ ^46^ ^]^ and, for instance, applied in the anodic bonding of silicon and glass substrates. Nevertheless, it must be assumed that changes in the local chemistry contribute to the local material displacement, although no change in chemical composition due to PEAR structuring has been detected so far.

However, silicon‐dioxide, for instance, showed after electron bombardment an increased etch rate in hydrofluoric acid (HF) by a factor of 3 (15 parts 49% HF, 10 parts 70% HNO_3,_ and 300 parts H_2_O),^[^ ^33^ ^]^ which is also well‐known for areas that underwent femtosecond laser irradiation with an increased etch rate by a factor of 50 as reported elsewhere.^[^ ^47^ ^]^ As a possible reason, a higher amount of 3‐member SiO_2_ ring structures (linked to the discussed D2 peak) with a lower Si‐O‐Si bond angle leads to a faster etch rate than higher cycle numbers in the silicate glass network.^[^ ^36^ ^]^ Although a difference in chemical composition of PEAR processed areas and pristine glass areas was not observed, as mentioned before, an increased etch rate from 20 nm s^−1^ (pristine areas) to 45 nm s^−1^ (PEAR with 155 µC µm^−^^2^) was also observed for PEAR structured areas using synthetic fused silica (Spectrosil2000) as target materials and a 50 vol% HF solution.

**Figure 4:**
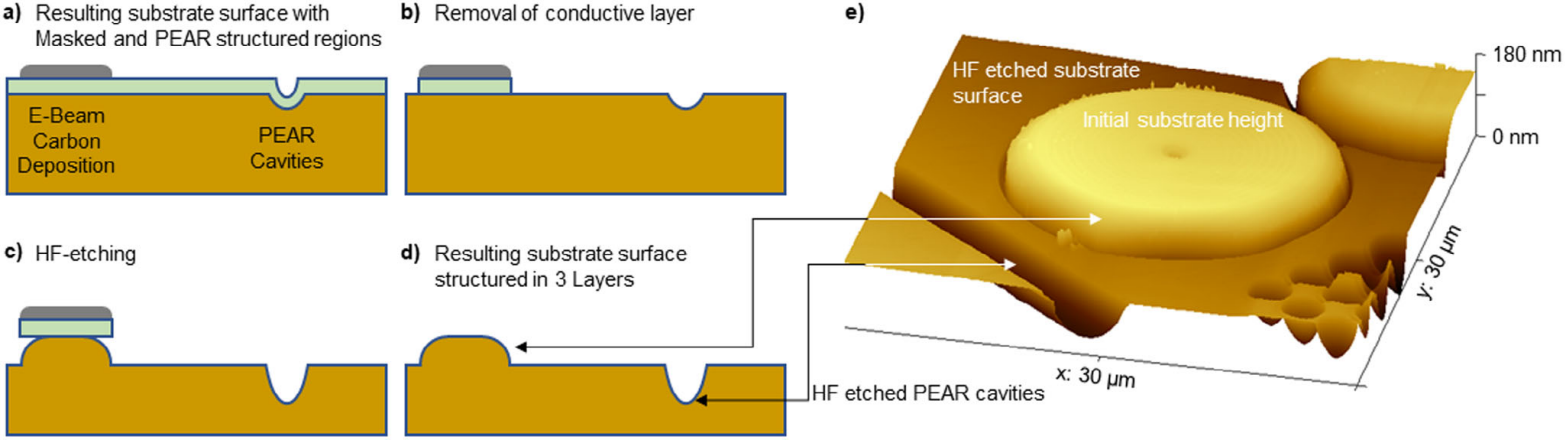
a–d) Schematic illustration of the designed 3‐level PEAR process for the future creation of more complex PEAR structuring including PEAR cavity formation and localized carbine deposition in an SEM, localized removal of the conductive top layer, and a subsequent HF wet‐etching to transfer the pattern into the glass surface. e) AFM surface scan of 3‐level PEAR structuring in synthetic fused silica (Spectrosil2000) within one final etch step (30 s, 50 vol% HF solution).

By combining this modified wet‐chemical etching behavior with a focused electron beam‐induced carbon deposition and PEAR structuring, a three‐level PEAR structuring process was designed to demonstrate the ability to even create more complex structures in the future moving toward 3D PEAR structuring. The concept is illustrated in **Figure** **4**e and was partially inspired by other techniques like metal‐assisted chemical etching (MaCE).^[^ ^48^ ^]^ Besides the PEAR cavities in synthetic fused silica (Spectrosil2000), localized carbon deposits from the chamber atmosphere^[^ ^49^ ^]^ were created on the Cr/Au conductive top layer using the recommended parameters for SEM images as mentioned before (930 µC µm^−^^2^, 10 kV, 0.086 nA). These locally created thin carbon deposits serve as a protective mask to preserve these areas during subsequent etching of the conductive metal top layer. Applying a subsequent HF treatment for 30 s results consequently in a localized etching of the glass surface. The deepest features are created in areas that underwent PEAR processing due to the initial existence of a cavity and the increased etch rate in these areas. Pristine glass areas that were neither exposed to PEAR nor localized carbon deposition represent the intermediate height level. Areas that are still masked by carbon and the conductive top layer are not directly etched in HF and represent therefore the top level of patterning. Nevertheless, the protective mask is etched under because of the isotropic nature of HF wet‐etching, which results in dome‐shaped structures. Such three‐level structures might be, for instance, useful for optical and microfluidic applications. Despite the novel technological possibilities offered by PEAR structuring, further elucidation of the PEAR mechanisms as well as an improved and validated PEAR model is required to unleash the full potential of PEAR structuring in the future that should also be transferable to other insulating materials.

## 3 Summary

Within the framework of this research, we were able to demonstrate the modifications in glass surfaces by focused electron beam irradiation in conventional SEMs but that can be exploited for the direct structuring of glasses. For this so‐called PEAR structuring, a variety of glass substrate materials and conductive top layer configurations were explored in dependence on the SEM beam parameters.

We showed that PEAR structuring takes place in the glass substrate and that the PEAR cavity depth depends significantly on the applied acceleration voltage. However, a saturation effect occurs above a dose of ≈200 µC µm^−^^2^. Furthermore, the achieved depth and diameter of the cavities are linked and depend on the material. For the parametric studies, synthetic fused silica (Spectrosil2000) was mainly used. To stay in the operation regime of conventional SEM, acceleration voltages between 5 and 15 kV were used. The impact of a conductive, charge‐dissipation layer was also studied, and we showed that thinner conductive coatings made from light‐weight elements, e.g. 5 nm carbon layer, sufficed yielding cavities with a diameter of ≈500 nm and a depth of over 200 nm during PEAR structuring. PEAR structuring therefore challenges the non‐destructive examination of glass samples with a focused electron beam. Therefore, one regime for PEAR patterning and one for SEM imaging were proposed. Although the underlying mechanisms of PEAR must be still further elucidated, electron beam‐induced surface charging, ion migration, and changes in the glass network appear as the major contributors. The chemical behavior during wet‐etching is also influenced. Surfaces patterned with PEAR showed a wet etch rate increased by a factor of about two in HF solution. The impact of the chemical composition on PEAR structuring was also evidently demonstrated. The largest PEAR cavity depth was achieved in the so‐called ULE glass which offers an extremely low thermal expansion coefficient. For borosilicates like D263 T and alumosilicates like AS 87 hillock formation was observed in dependence on the exposure parameters, which was attributed to the high amount of mobile alkaline ions. By combining PEAR structuring with a localized deposition of carbon in the SEM and the different wet chemical behavior of PEAR structured and pristine glass surfaces in HF solution, a three‐level structuring process could be demonstrated, which enables for fabrication of new degrees of freedom conceivable, e.g. for the field of optics and microfluidics. Furthermore, the embedding of metal structures in a glass surface, the generation of QR codes and graphic elements in a glass surface as well as the structuring of 3D glass structures were demonstrated. PEAR structuring thus represents a fascinating direct structuring method and a novel possibility for structuring glasses, which is practically feasible in any conventional SEM.

## 4 Experimental Section

The raw data associated with the images are provided accordingly in figshare:

Figure 1: 10.6084/m9.figshare.23103962

Figure 2: 10.6084/m9.figshare.23128085

Figure 3: 10.6084/m9.figshare.23103947

Figure 4: 10.6084/m9.figshare.23103851

**Figure 5:**
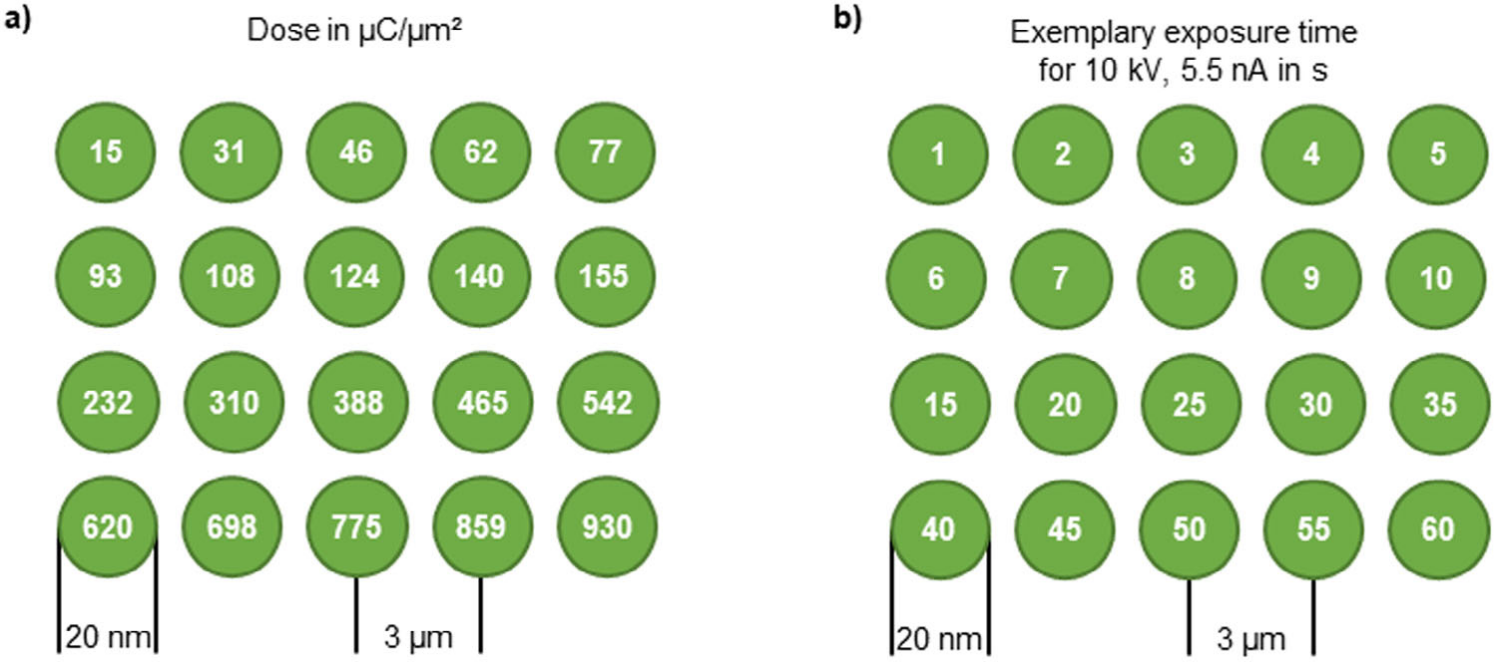
Schematic illustration of the used standard test pattern constating of a 4 × 5 arrangement of beam exposure circles: a) illustration of the dose variation within the array and b) variation of the dose with respect to the exposure time the specified dose of (a). For the PEAR experiments, the electron acceleration voltages and electron emission currents varied from 5 to 15 kV and 680 to 22 nA, respectively. It should be noted that the dose variation is always kept constant (cf. Figure 5a) for each set of parameters. Therefore, exposure times need to be adjusted according to the applied electron emission current. The exposure time for 10 kV and 5.5 nA is exemplarily given in Figure 5b.

PEAR patterning was mostly realized in a Helios 600i from FEI and features were defined in the pattern editor of a FEI Helios Nanolab 600i and subsequently written utilizing electron beam mode. To explore PEAR structuring, a standard test pattern was created that consists of an array based on a 4 × 5 arrangement of circular features (diameter 20 nm) as shown in **Figure** **5**a. The distance between the circle centers was always 3 µm. A complete array was always linked to a fixed electron acceleration voltage and emission current, but the dose however varies for each circular array element from 15 and 930 µC µm^−^^2^ (Figure 5a). The dose could be set by adjusting the beam current and the exposure time (Figure 5b). The dwell time was 1 µs with a beam diameter of 5 nm at 5.5 nA, an overlap in X & Y of 50%, and a pitch X/Y of 2.5 nm. The scan type for patterning was set to serpentine with a solid fill style and a scan direction from bottom to top.

The SEM chamber was preconditioned by pumping at least 16 h after venting. As the sample exchange requires full venting of the chamber (no load lock equipped) the chamber was cleaned with an inbuilt plasma cleaner once a week. The vacuum system consists of a nitrogen purged backing pump (oil‐free dry scroll pump Edwards XDS10) connected to the chamber via a turbopump (Pfeiffer HiPace300Plus). The electron gun and the column were constantly pumped by two ion getter pumps, another ion getter pump was pumping the chamber from a pressure below 10^−5^ mbar. The AFM images and line profile shown were performed on a Cypher from Asylum Research in AC mode. For the determination of depth and FWHM, only raw data along the scan direction were used.

For experiments shown in this publication, if not otherwise specified, synthetic fused silica (Spectrosil2000) substrates 10 mm × 10 mm × 550 µm in size that were coated by electron beam evaporation (PVD‐Cluster Ardenne CS400ES) with a 10 nm chromium and 50 nm gold conductive top layer were used. The tooling base pressure of <5 × 10^−7^ mbar was achieved with a Pfeiffer turbopump TPH1201PN, during the evaporation the pressure rises to the 10^−6^ mbar regimes. The acceleration voltage was 10 kV the current depends on the material and was adjusted to achieve the desired deposition rate. Due to the multi‐hearth e‐beam source EVM10 from FerroTec, multiple metal targets could be used for the evaporation process without breaking the vacuum. For example, Cr/Au was deposited in situ at a rate of 0.02/0.05 nm s^−1^ with a current *I*_E_ = 20/26 mA. The sample was kept at ≈20 °C room temperature and rotated at a rate of 10 rotations per minute. The nominal thickness was controlled with a thin‐film thickness monitor SQM160 and a quartz crystal microbalance. The specific tooling factors were regularly calibrated with lift‐off samples and profilometric measurements.

Further conductive metal top layers were also deposited on Spectrosil2000 by electron beam evaporation including Cr/Al, Al, Cr, Cr/W, W, Ti, and Fe. Ultra‐thin Cr and C layers of ≈5 nm were created by means of a Gatan precision etching and coating system (Model PECS 682).

The PEAR‐Process was studied using various glass bulk materials including AS87 and D263T (from Schott), ULE7972 (from Corning), and HQQ310, INF302, SUP312, SUP300, and SUP1 (from Heraeus). A short overview of their respective characteristics is given in Table 1.

## Conflict of Interest

The authors declare no conflict of interest.

## Author Contributions

M.H., M.H., and C.W. performed the experiments and carried out the measurements, M.H., M.H., C.W., S.S. discussed the research and wrote the manuscript, and S.S. supervised the entire research and finalized the manuscript.

## Supporting information



Supporting Information
