# Supplementary material for: Advancing Glass Engineering: Harnessing Focused Electron Beams for Direct Microstructuring
Source: Small Methods. 2025 Mar 9;9(5):2401671. doi: 10.1002/smtd.202401671 (PMC12103231; doi:10.1002/smtd.202401671)
Supplement: Supplementary file 1 — Supporting Information [file SMTD-9-2401671-s001.pdf]

small
methods

Supporting Information

for *Small Methods*, DOI 10.1002/smtd.202401671

Advancing Glass Engineering: Harnessing Focused Electron Beams for Direct Microstructuring

Mathias Holz, Martin Hofmann, Christoph Weigel and Steffen Strehle**

Supplementary Information

Advancing Glass Engineering: Harnessing Focused Electron Beams for Direct Microstructuring

Mathias Holz, Martin Hofmann, Christoph Weigel, Steffen Strehle

Institute of Micro- and Nanotechnologies MacroNano®, Microsystems Technology Group, Technische Universität Ilmenau, Ilmenau, Germany

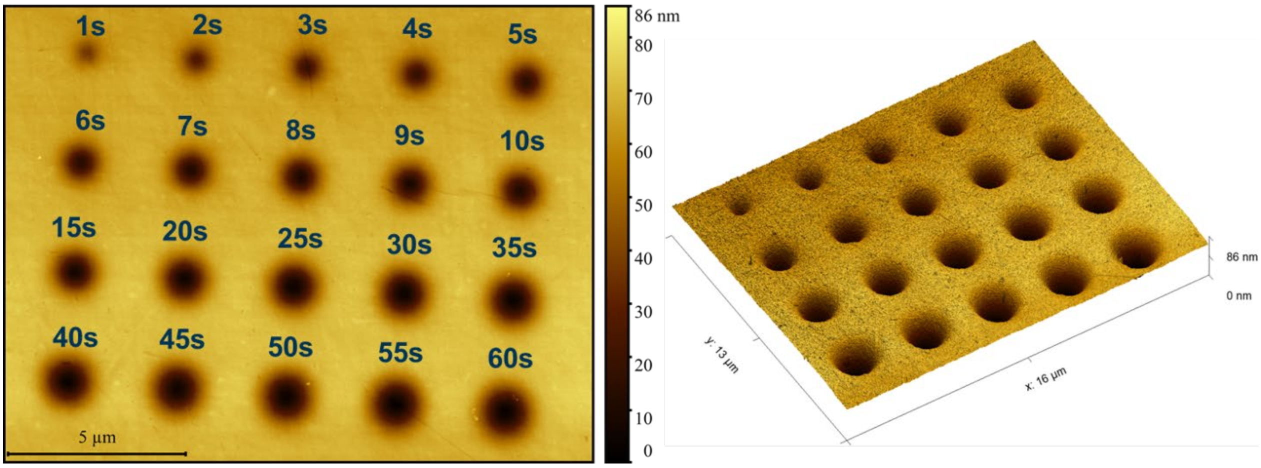

[Figure S1]: AFM surface image of the standard test pattern as shown in Figure 5 used for the PEAR structuring of synthetic fused silica (Spectrosil2000). The glass sample was covered with a film stack consisting of 10 nm Cr and 50 nm Au serving as a conductive top coating. PEAR process parameters: 10 kV, 5.5 nA, dose from 15 µC/µm² to 930µC/µm², circular exposure area diameter 20 nm.

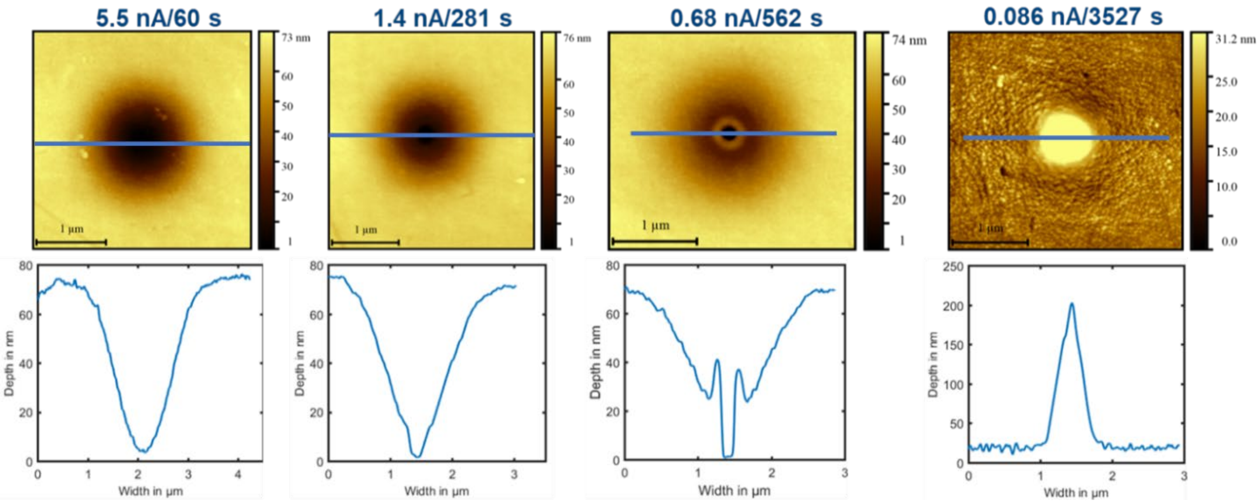

[Figure S2]: AFM surface image and extracted line profile as indicated (blue line) of PEAR cavities created with varying electron beam current at 10 kV, 930 µC/µm². The

beam current was varied from 5.5 to 0.086 nA while keeping the circular exposure area (diameter 20 nm) constant. As shown in the manuscript, depth saturation is reached at doses higher than 232 µC/µm², which is also visible for beam currents of 5.5 nA and 1.4 nA. At a beam current of 0.086 nA, only hillock formation is visible, most likely due to carbon deposition from the SEM chamber atmosphere.

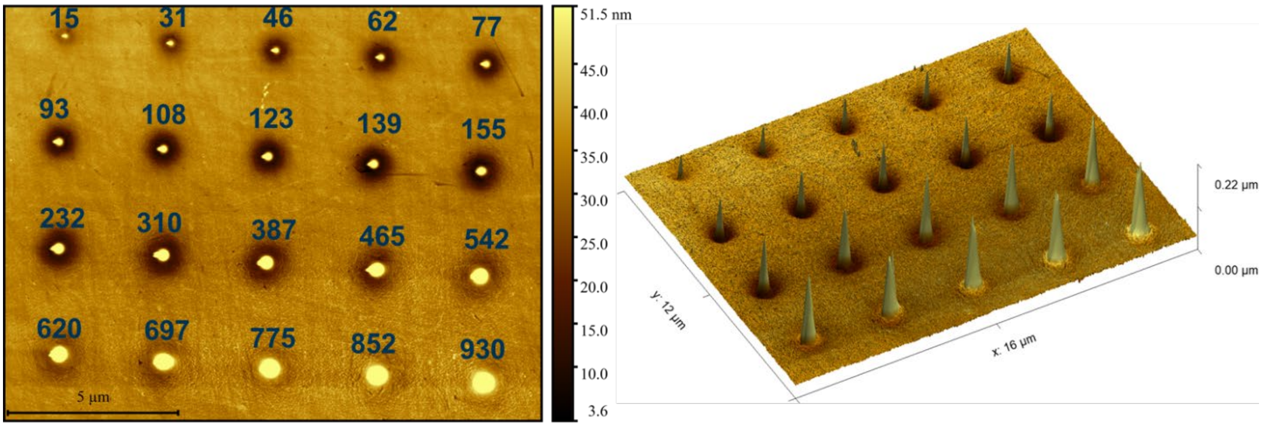

[Figure S3]: AFM image of the standard test pattern written on synthetic fused silica (Spectrosil2000) covered by a 10 nm Cr / 50 nm Au conductive coating. PEAR process parameters: 10 kV, 0.086 nA, dose from 15 µC/µm² to 930 µC/µm². The experiment explores the dose dependency for the previously determined deposition regime [S2 right images]. It shows that also for a lower dose < 775 µC/µm² a compensation effect of shallow PEAR structuring and hillock formation appears to be present as it was also recognized in Figure 3 f and e for AS87 and D263T.

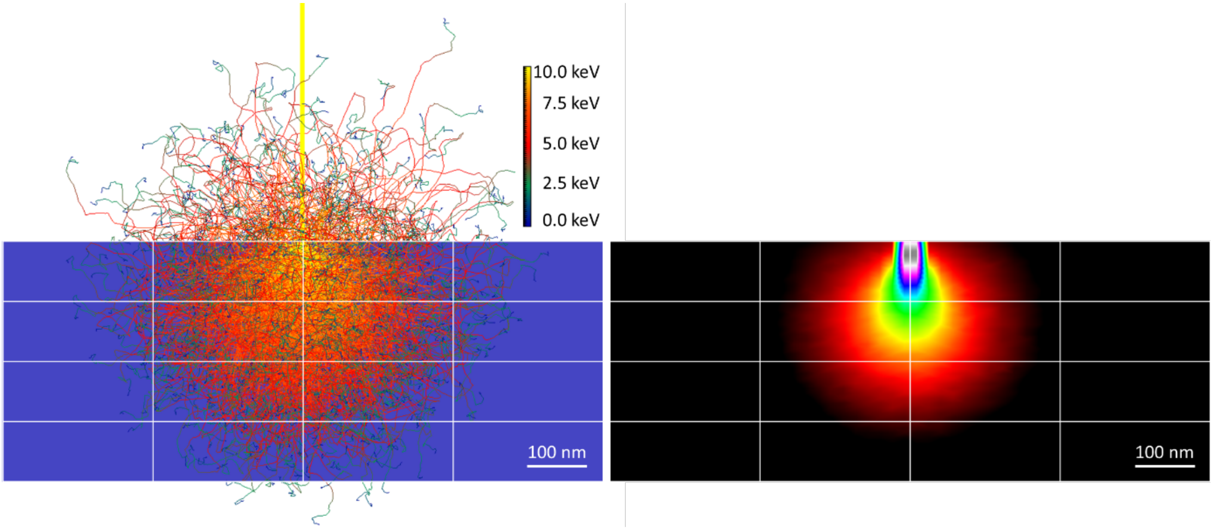

[Figure S4] Monte Carlo simulation of electron trajectories generated with CASINO software. Box size 250 nm x 100 nm. The simulated beam diameter is 5 nm with an energy of 10 keV. The sample is 400 nm thick chromium layer. The trajectories are colored depending on their respective energy, the cut-off energy is 0.05 keV, 1000 trajectories are displayed while 100000 electrons were simulated.

Reference of CASINO software:

H. Demers, N. Poirier-Demers, A. Réal Couture, D. Joly, M. Guilmain, N. de Jonge, and D. Drouin. (2011). "Three-dimensional electron microscopy simulation with the CASINO Monte Carlo software." Scanning 33(3): 135-146.

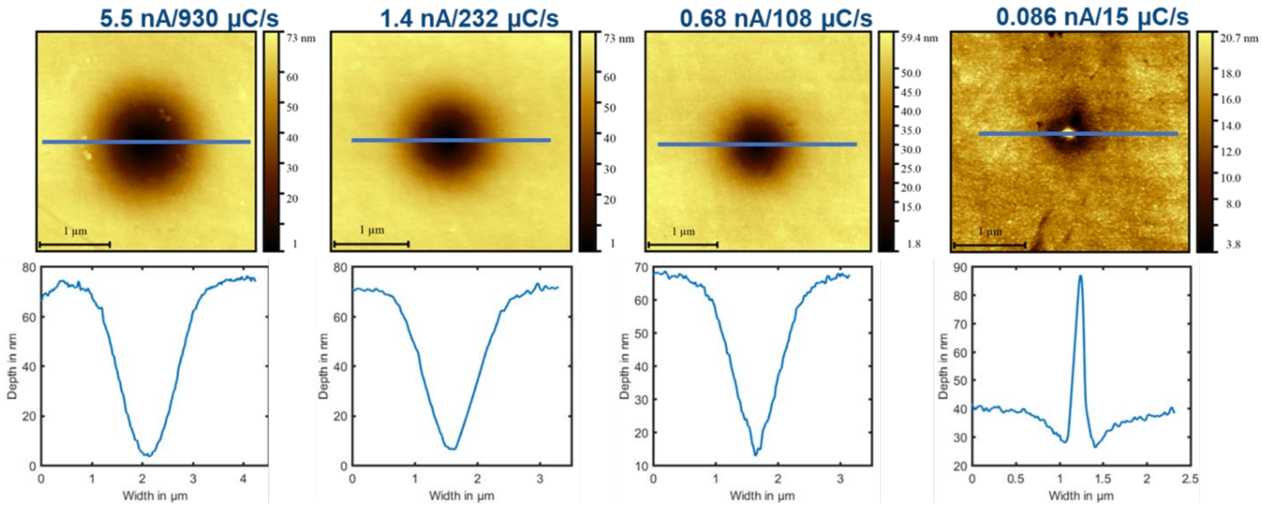

[Figure S5]: AFM surface image and line profile as indicated (blue line) of PEAR cavities created with varying beam current at 10 kV acceleration voltage using a constant exposure time of 60 s, the dose varies accordingly. A lower beam current tends to create suspected carbon deposition.

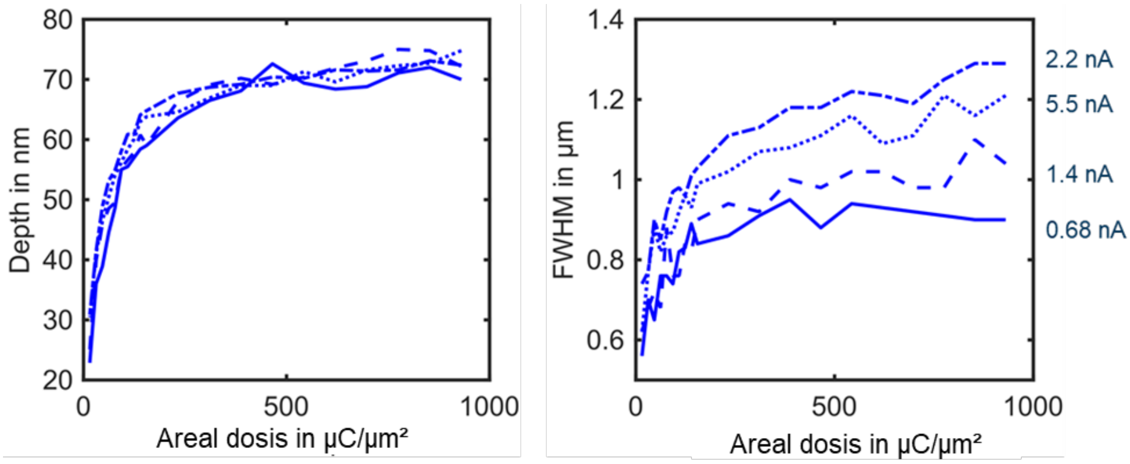

[Figure S6]: Depth and FWHM of PEAR cavities in dependence on the beam current at a constant acceleration voltage of 10 kV. The cavity depth appears independent on the set emission current.

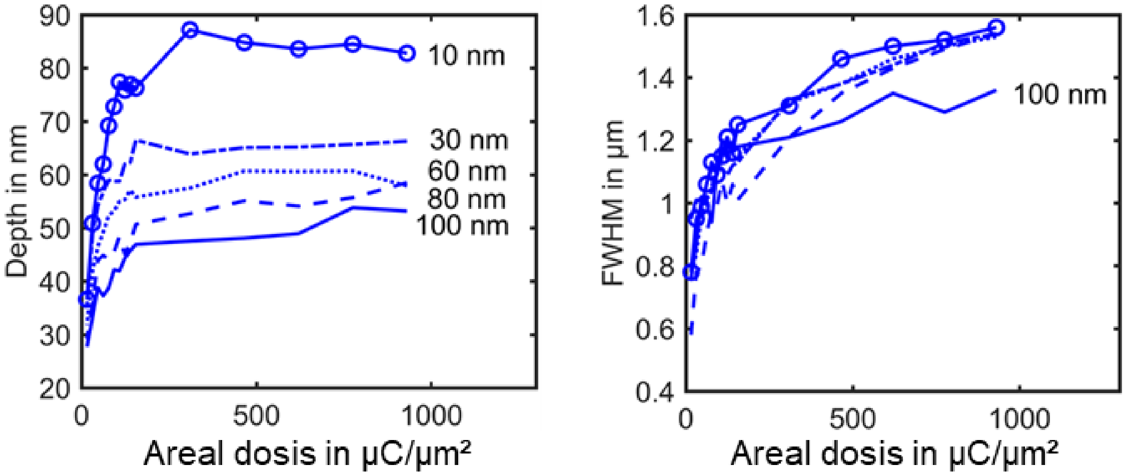

[Figure S7] Dependence of the PEAR cavity depth created at 10 kV acceleration voltage and 5.5 nA beam current on the Cr top-layer thickness. Thinner layers tend to promote deeper cavities, while the width (FWHM) remains hardly affected, especially in the range 10 to 80 nm Cr thickness.

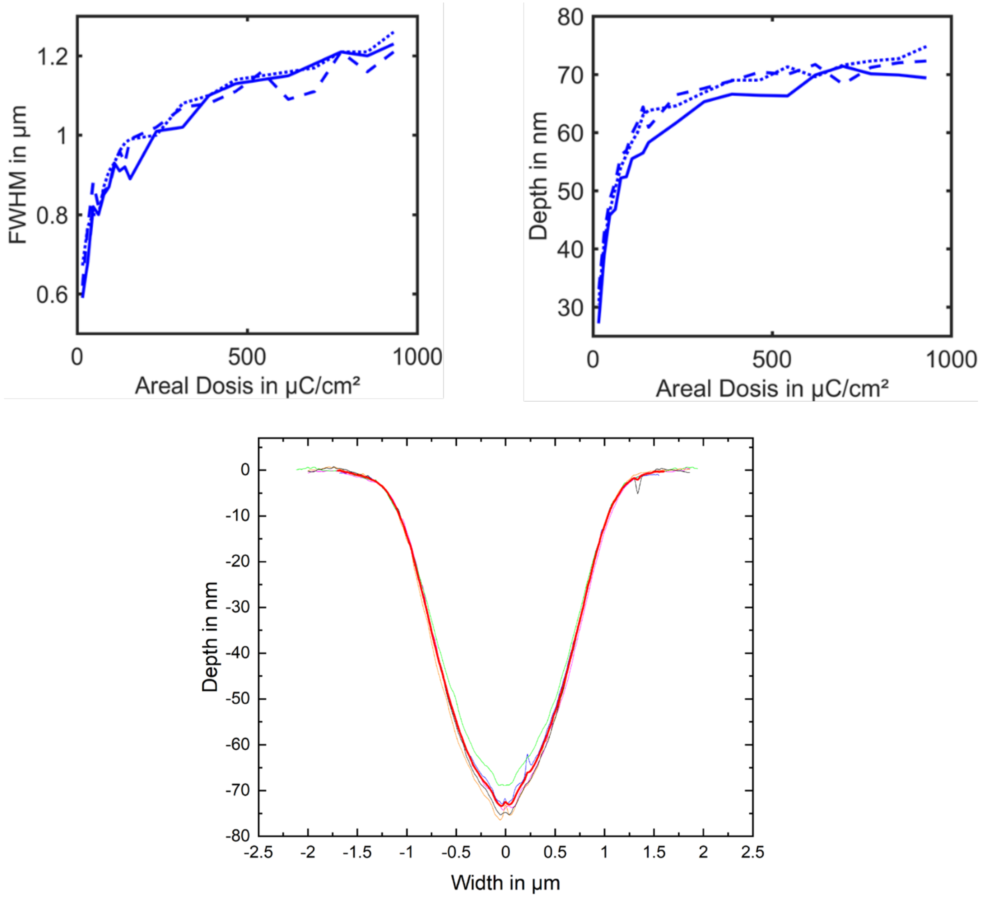

[Figure S8] (up left) and (up right) shows the evaluation of FWHM and depth for three identically patterned structures (10 kV, 5.5 nA) on Spectrosil2000, coated with 10 nm / 50 nm Cr/Au as conductive layer. The analysis addresses patterning stability, AFM-specific measurement errors, and manual determination inaccuracies for depth and FWHM. The results demonstrate a reproducibility of ±5 nm in depth and ±50 nm in

width. The image down shows five profiles extracted from AFM measurements of five identical patterns written on a single sample (Spectrosil2000, 30 nm Cr conductive layer, 10 kV, 5.5 nA) with a dose of 775 µC/µm². The sixth, red line represents the averaged profile. The image evaluates patterning stability and potential AFM measurement errors, such as profile extraction inconsistencies or tip-specific deviations.

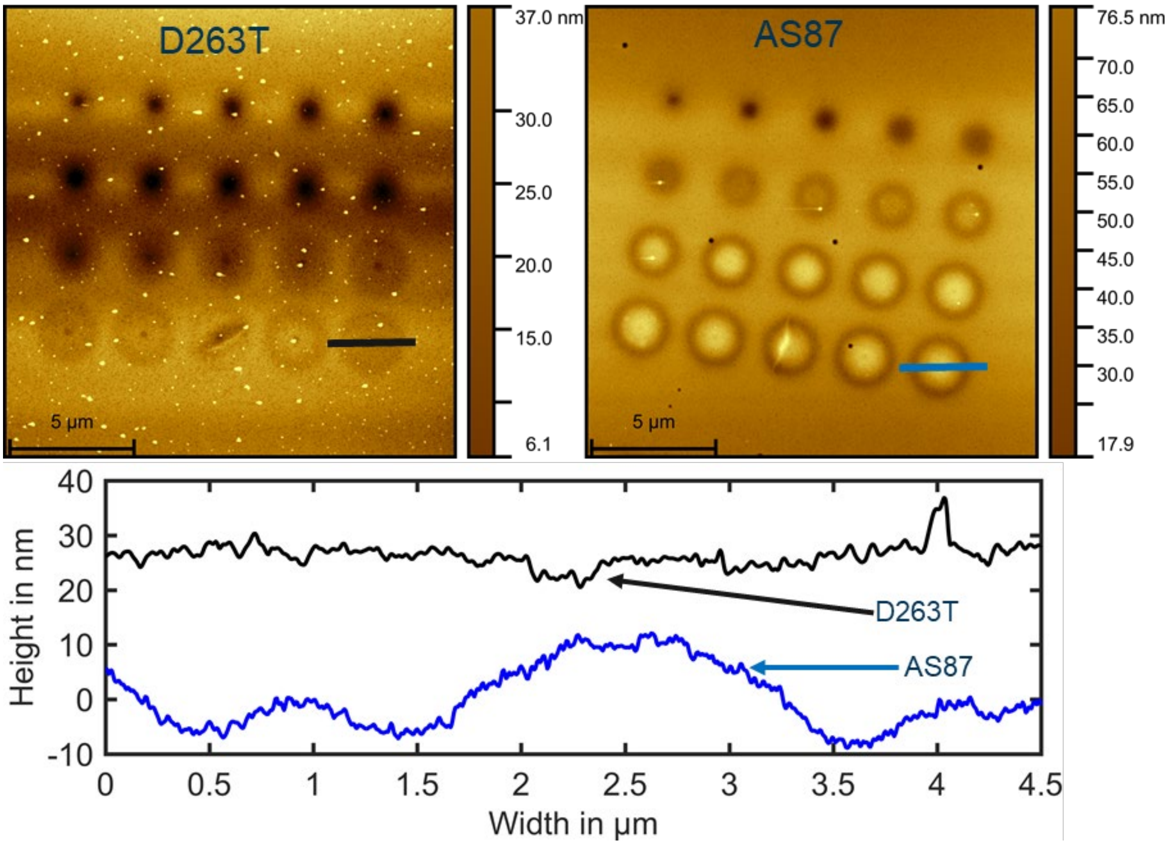

[Figure S9] Hillock formation within the standard exposure pattern (dose from 15 µC/µm² to 930 µC/µm²) on D263T and AS87 covered by a 30 nm Cr structured conductive top-coating. PEAR parameters: 10 kV, 5.5 nA.

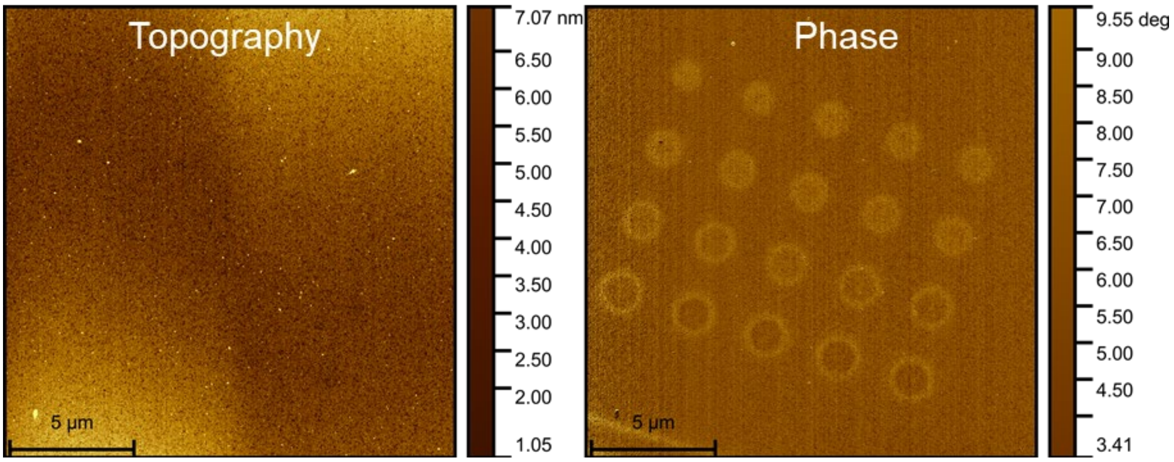

[Figure S10] Standard exposure pattern (dose from 15 µC/µm² to 930 µC/µm²) created by PEAR structuring on a sapphire substrate. PEAR parameters: 10 kV, 5.5 nA, 30 nm Cr conductive top-coating. There is no cavity or hillock formation detectable in the topography signal. However, the structures are clearly visible in the phase image. This still could indicate additional electrostatic forces (for example, trapped charges or ionizations) that cause a phase shift without a change in topography.

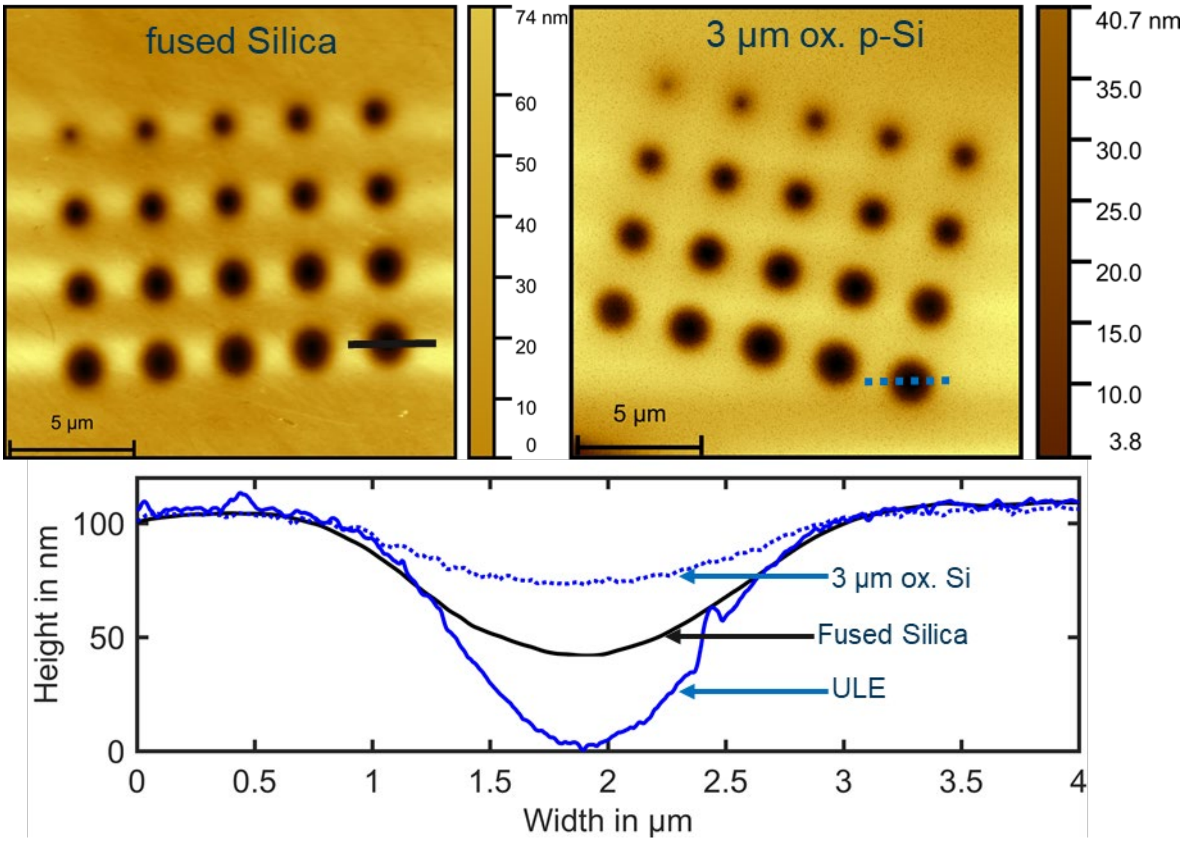

[Figure S11] Comparison of fused silica (Spec2000), wet oxidized 3 µm p-Si and ULE. Thermally oxidized silicon is clearly possible to be structured, underlining silicon or oxygen as key driver for the PEAR processing. Whereas titanium (network former in ULE) might strengthen the process.

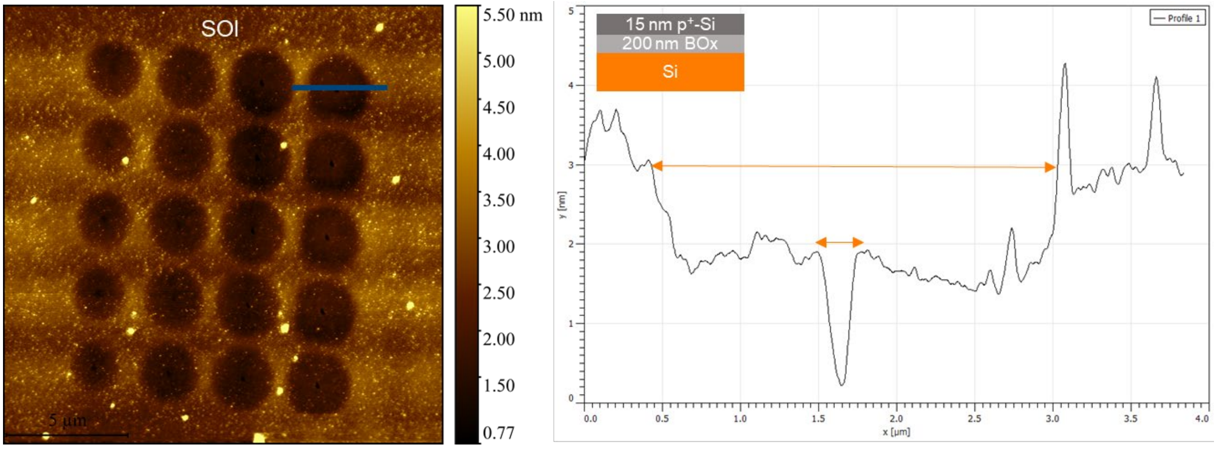

[Figure S12] Standard pattern (dose from 15 µC/µm² to 930 µC/µm²) on silicon-on-insulator substrates (Si: silicon, BOx: silicon dioxide layer) structured at 10 kV and 5.5 nA, indicating that PEAR structuring is also possible for thin buried oxide layer without further conductive coating.

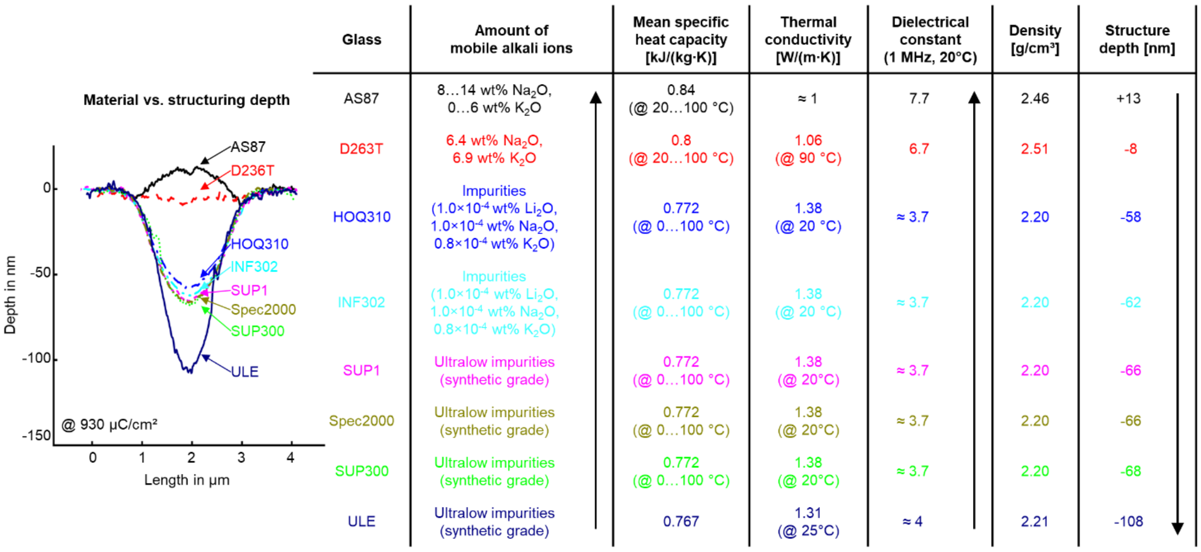

Glass	Amount of mobile alkali ions	Mean specific heat capacity [kJ/(kg·K)]	Thermal conductivity [W/(m·K)]	Dielectrical constant (1 MHz, 20°C)	Density [g/cm³]	Structure depth [nm]
AS87	8…14 wt% Na_2O, 0…6 wt% K_2O	0.84 (@ 20…100 °C)	≈ 1	7.7	2.46	+13
D263T	6.4 wt% Na_2O, 6.9 wt% K_2O	0.8 (@ 20…100 °C)	1.06 (@ 90 °C)	6.7	2.51	-8
HOQ310	Impurities (1.0×10^{-4} wt% Li_2O, 1.0×10^{-4} wt% Na_2O, 0.8×10^{-4} wt% K_2O)	0.772 (@ 0…100 °C)	1.38 (@ 20 °C)	≈ 3.7	2.20	-58
INF302	Impurities (1.0×10^{-4} wt% Li_2O, 1.0×10^{-4} wt% Na_2O, 0.8×10^{-4} wt% K_2O)	0.772 (@ 0…100 °C)	1.38 (@ 20 °C)	≈ 3.7	2.20	-62
SUP1	Ultralow impurities (synthetic grade)	0.772 (@ 0…100 °C)	1.38 (@ 20°C)	≈ 3.7	2.20	-66
Spec2000	Ultralow impurities (synthetic grade)	0.772 (@ 0…100 °C)	1.38 (@ 20°C)	≈ 3.7	2.20	-66
SUP300	Ultralow impurities (synthetic grade)	0.772 (@ 0…100 °C)	1.38 (@ 20°C)	≈ 3.7	2.20	-68
ULE	Ultralow impurities (synthetic grade)	0.767	1.31 (@ 25°C)	≈ 4	2.21	-108

[Figure S13] Dependence of the patterning depth for different glasses with different amounts of (mobile) alkali ions and associated dielectric constants. Glasses with a high amount of alkaline ions show higher dielectric constants, mean specific heat capacities and densities as well as lower structure depths up to an inverse behavior (e.g. alumosilicate glass AS87).

References for glass compositions / impurities and material properties:

- ULE (synthetic grade, low metallic impurities)
    - Carapella, A. P., Duran, C. A., Hrdina, K. E., Sears, D. J. & Tingley, J. E. ULE® Glass for EUVL applications, a fictive temperature correlation. Journal of Non-Crystalline Solids **367,** 37–42; 10.1016/j.jnoncrysol.2013.01.052 (2013).
    - Corning, ULE® Corning Code 7972 Ultra Low Expansion Glass: Product information, 2016. https://www.corning.com/media/worldwide/csm/documents/7972%20ULE%20Product%20Information%20Jan%202016.pdf (accessed 21 September 2023).
    - Präzisions Glas & Optik GmbH. ULE® Ultra Low Expansion Titanium Silicate glass 7972. Available at https://www.pgo-online.com/intl/ule.html (2023).

- SUP1, Spec2000, SUP300 (synthetic grade, metallic impurities < 300 ppb)
    - Heraeus Quarzglas GmbH & Co. KG & Heraeus Conamic. Fused Quartz and Fused Silica for Optical Applications - Data and Properties. Available at https://www.heraeus.com/media/media/hca/doc_hca/products_and_solutions_8/optics/Data_and_Properties_Optics_fused_silica_EN.pdf (2023).
    - UQG (Optics) Ltd. Heraeus HOQ310. Quartz Glass for Optics: Data and Properties. Available at https://www.uqgoptics.com/wp-content/uploads/2019/03/Heraeus-HOQ310.pdf (2019).
- INF302, HOQ310 (typical trace impurities for Infrasil / HOQ)
    - UQG (Optics) Ltd. Heraeus HOQ310. Quartz Glass for Optics: Data and Properties. Available at https://www.uqgoptics.com/wp-content/uploads/2019/03/Heraeus-HOQ310.pdf (2019).
- D263T
    - Thiénot, E., Domingo, F., Cambril, E. & Gosse, C. Reactive ion etching of glass for biochip applications. Composition effects and surface damages. Microelectronic Engineering **83,** 1155–1158; 10.1016/j.mee.2006.01.029 (2006).
    - Schott AG. D 263® T eco. Datasheet. Available at https://www.schott.com/de-de/products/d-263-p1000318/downloads (2023).
    - Schott AG. Technical Glasses. Physical and Technical Properties. Available at https://www.schott.com/en-nl/expertise/glass-melting-and-hot-forming, accessed 21 September 2023 (2020).
- AS87 eco
    - Panzner, G., Freitag, R., Neupert, G. & Lautenschlaeger, G. EP2869989B1: BULLET-RESISTANT LAMINATED GLASS. Available at https://data.epo.org/gpi/EP2869989B1 (2013).
    - Schott AG. SCHOTT AS 87 eco. Datasheet. Available at https://www.schott.com/de-de/products/as-87-eco-p1000312/downloads (2021).
    - Präzisions Glas & Optik GmbH. AS 87 eco Dünnstglas | Ultradünnes Aluminosilikatglas. Available at https://www.pgo-online.com/de/as87.html (2023).

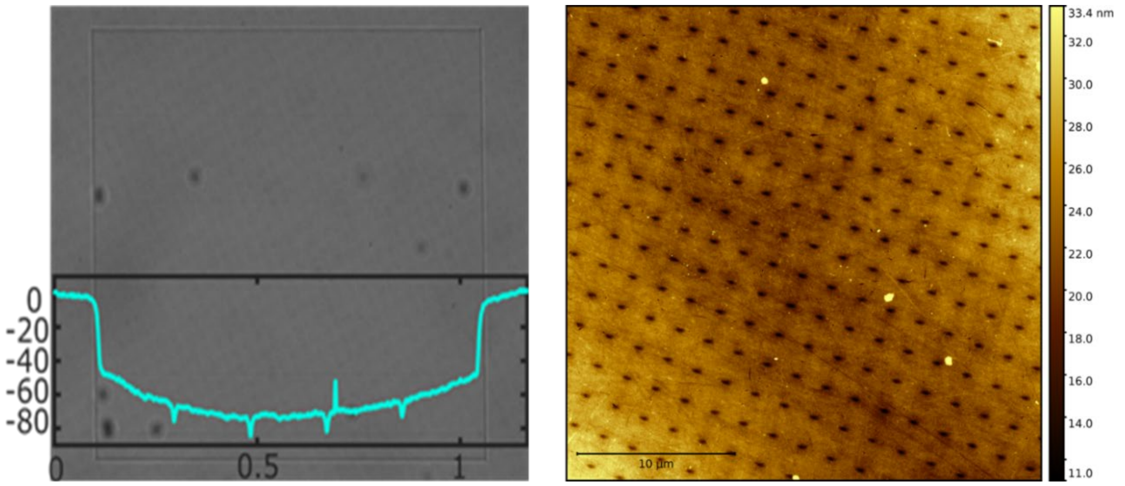

[Figure S14] (left) Optical image of a patterned 1 × 1 mm² field with subsequent metal removal. The SEM is operated in the FieldFreeMode, averages 4 Frames in FastScan, FullFrame. Patterned at 30 kV, 5.5 nA for 10 h in imaging mode of the SEM with measured Dektak profile (blue line). Verification of PEAR-structured cavities with a different contact measurement method using Veeco's Tactile profilometer Dektak 150. (right) The large field, in turn, is structured with 14.5M points. The dots have a depth of 10 nm and a width of 220 nm (FWHM). A section can be seen in the right AFM image. The point features show the capabilities of PEAR structuring in multiple layer heights in one step. The large field is used for further analysis [S12].

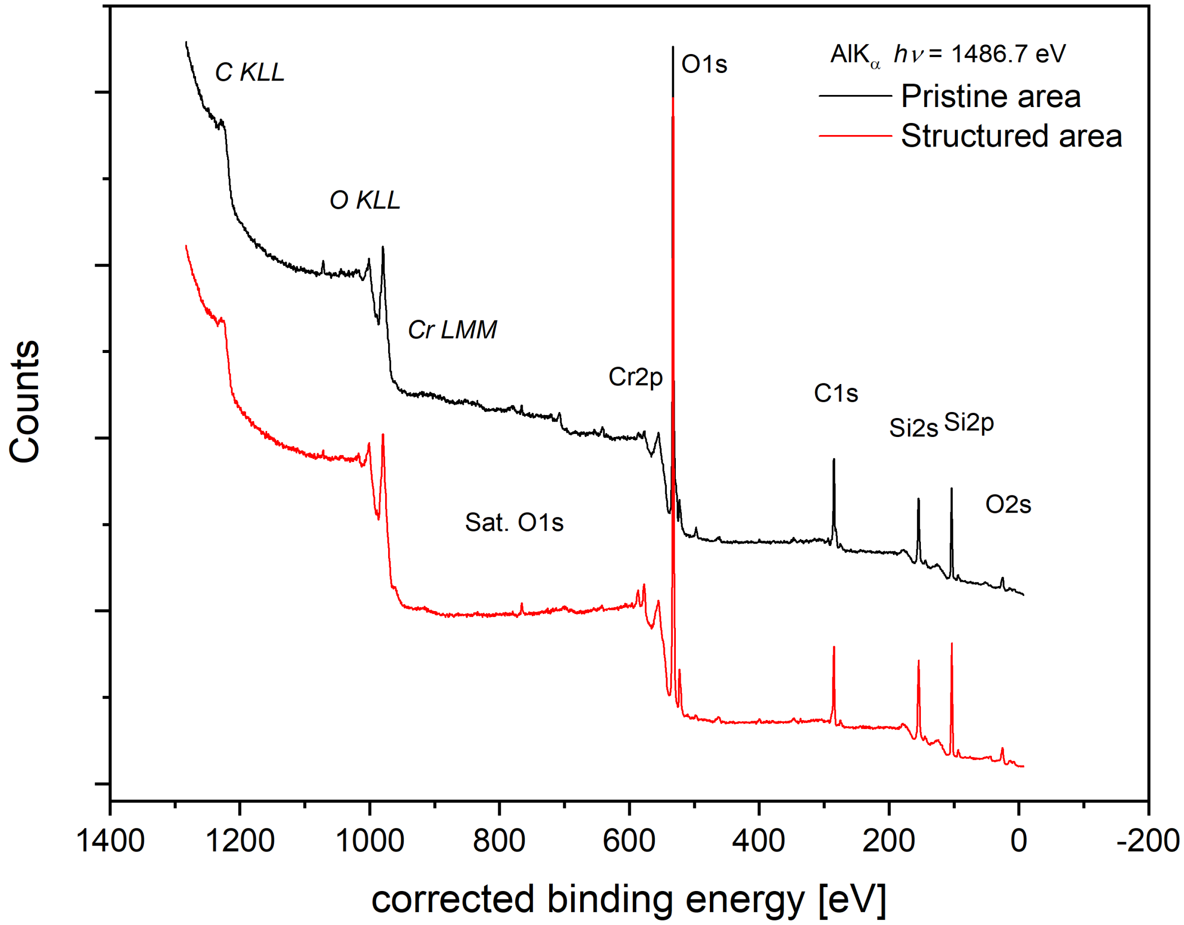

[Figure S15] XPS measurement within a PEAR structured and a pristine area of [S14] to explore a reduction of the oxygen concentration that might occur during PEAR structuring. The determination of the composition was based on high-resolution measurements of the O1s and the Si2p peaks (not shown). For the PEAR structured areas, the Si:O ratio is not decreased and appears even slightly increased by 8.5 at.% (Si:O ratio in pristine area 1:1.29 to Si:O ratio in structured area 1:1.4). The X-ray photoelectron spectroscopy (XPS) investigations were carried out using a SPECS SAGE HR 150 system with a 1D delayline detector using monochromatized AlKα radiation (excitation energy 1486.7 eV). The calibration of the energy scale was done by reference measurements on a polycrystalline silver sample. Investigation was done for the electrons emitted perpendicular to the surface (emission angle = 0°) using a constant analyzer energy (CAE mode). A constant fitting energy of 35 eV (n.-mon. AlKα) was used for the overview spectra and 13 eV for the energetically high-resolution detail spectra (XPS monochromatic AlKα: nominal resolution of the Ag3d5/2 state < 0.75 eV at the measurement settings used). No charge neutralization was performed during the measurement. The analysis areas had a diameter of 1 mm, which correspond to the PEAR structured area (1×1 mm²).

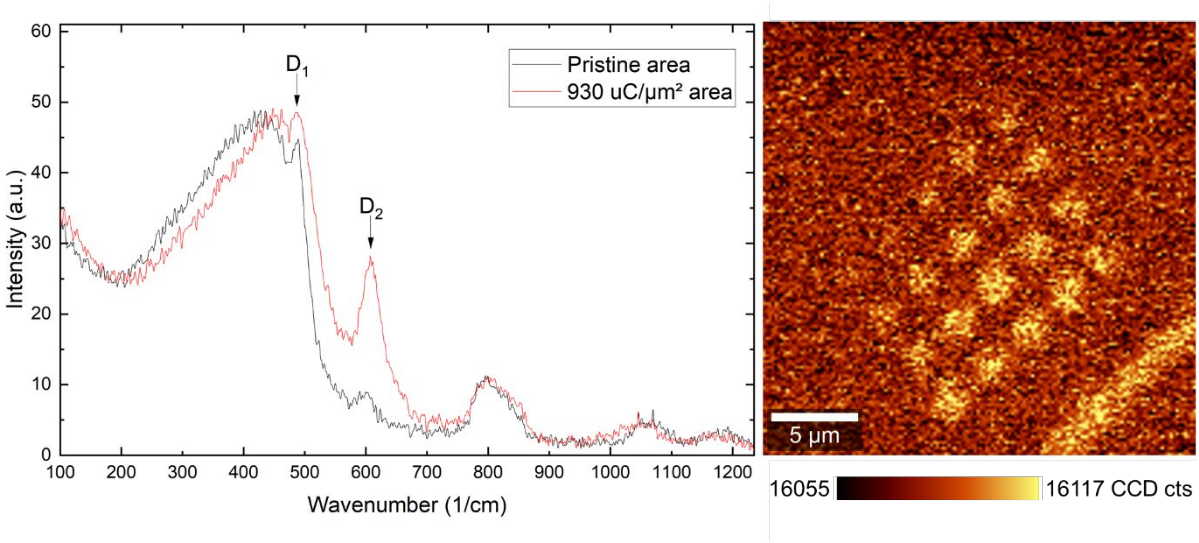

[Figure S16] Left image: Raman spectroscopy measurement of a pristine area and a PEAR structured area, structured with 930 µC/µm²; Right image: Raman spectroscopy measurement as surface mapping at 605 1/cm revealing the PEAR structured pattern.

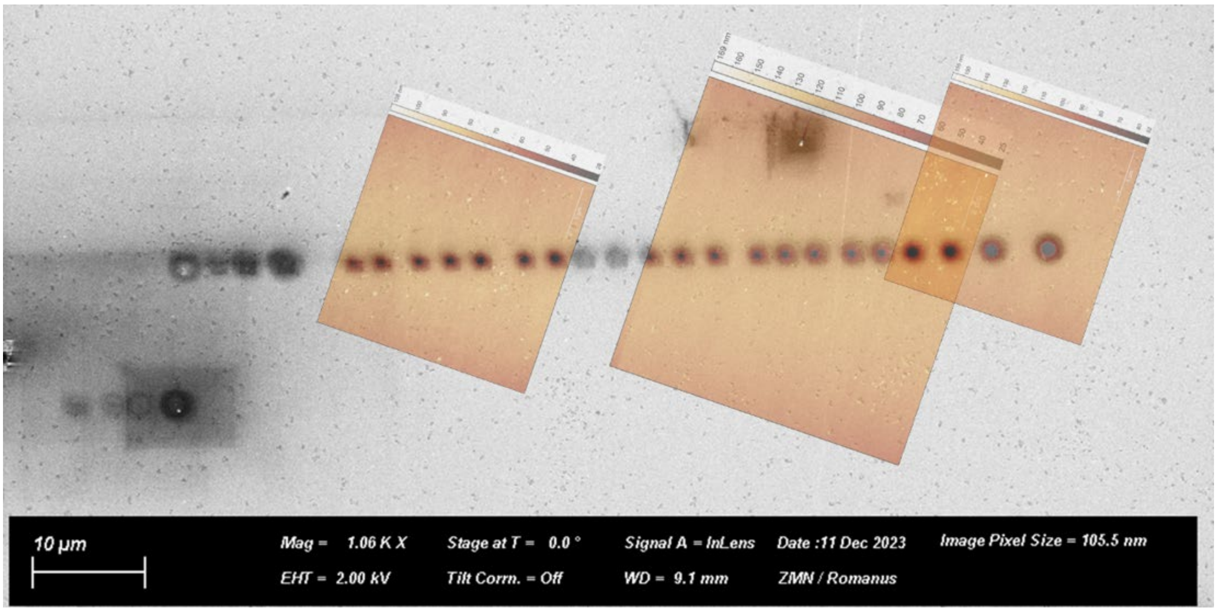

[Figure S17] SEM image of PEAR structuring realised with an Auriga 60 SEM from Zeiss with an additional overlay of the according AFM images proving cavity formation due to the PEAR structuring. We also used the EDX mode of a Hitachi 4800 to scan lines and generate points (not shown).

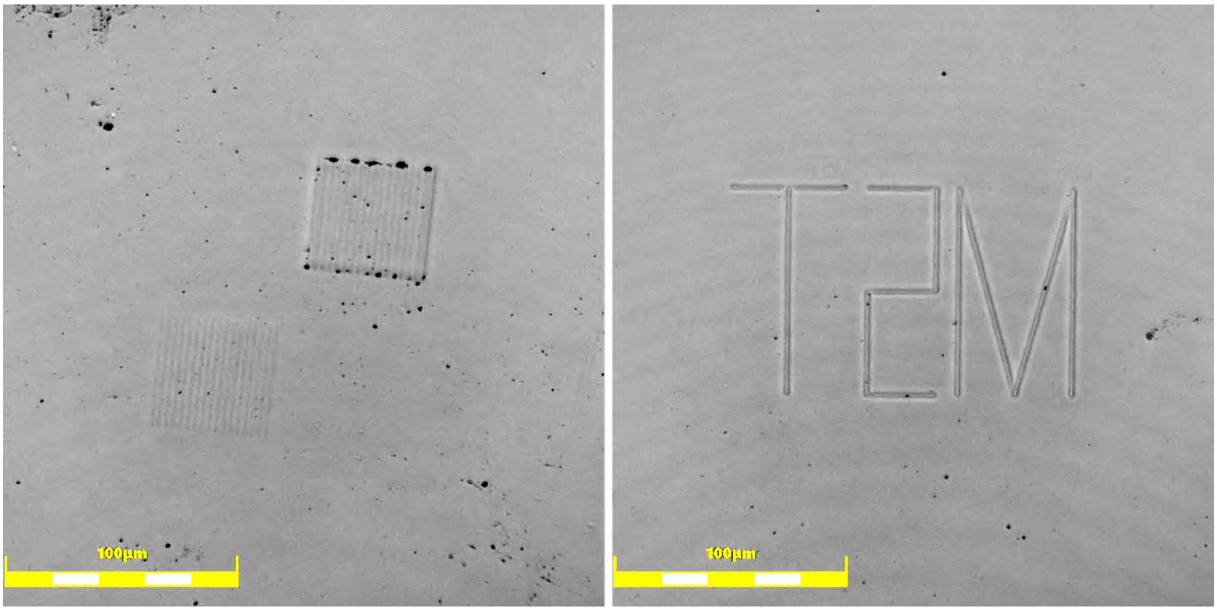

[Figure S18] Soft stamp (PDMS) created from a of PEAR structured glass master to be used in nanoimprint lithography. Features from Figure 1d and e have been imprinted to exclude trapped charges as possible reason for the gained images.
